# Comprehensive analysis of morphology, transcriptomics, and metabolomics of banana (*Musa* spp.) molecular mechanisms related to plant height

**DOI:** 10.3389/fpls.2025.1509193

**Published:** 2025-03-25

**Authors:** Junya Wei, Guoyin Liu, Mingzhen Sun, Hao Wang, Ping Yang, Shimin Cheng, Lina Huang, Shouxing Wei, Debing Liu

**Affiliations:** ^1^ Tropical Crops Genetic Resources Institute, Chinese Academy of Tropical Agricultural Sciences/National Key Laboratory for Tropical Crop Breeding/National Tropical Fruit Improvement Center/Key Laboratory of Crop Genetic Resources and Germplasm Creation in South China, Ministry of Agriculture and Rural Affairs/Key Laboratory of Genetic Improvement and Innovation of Tropical Crop Resources of Hainan Province, Haikou, Hainan, China; ^2^ Tropical Agriculture and Forestry College, Hainan University, Haikou, Hainan, China; ^3^ School of Design, Hainan Vocational University of Science and Technology, Haikou, Hainan, China

**Keywords:** banana, metabolomics, transcriptomics, hormones, height

## Abstract

**Introduction:**

Plant height is an important agronomic trait that not only affects crop yield but is also related to crop resistance to abiotic and biotic stresses.

**Methods:**

In this study, we analyzed the differentially expressed genes (DEGs) and differentially accumulated metabolites (DAMs) between Brazilian banana and local dwarf banana (Df19) through transcriptomics and metabolomics, and combined morphological differences and endogenous hormone content to analyze and discuss themolecular mechanisms controlling banana height.

**Results:**

Sequencing data showed that a total of 2851 DEGs and 1037 DAMs were detected between Brazilian banana and local dwarf banana (Df19). The main differential biological pathways of DEGs involve plant hormone signaling transduction, Cutin, suberin and wax biosynthesis, phenylpropanoid biosynthesis, mitogen-activated protein kinase (MAPK) signaling pathway in plants, amino sugar and nucleotide sugar metabolism, etc. DAMs were mainly enriched in ATP binding cassette (ABC) transporters, amino and nucleotide sugar metabolism, glycerophospholipid metabolism, lysine degradation, and phenylalanine metabolism.

**Discussion:**

Our analysis results indicate that banana plant height is the result of the synergistic effects of hormones such as abscisic acid (ABA), gibberellic acid (GA3), indole-3-acetic acid (IAA), jasmonic acid (JA), brassinosteroids (BR) and other plant hormones related to growth. In addition, transcription factors and ABC transporters may also play important regulatory roles in regulating the height of banana plants.

## Introduction

The height of crops is an important and valuable agronomic trait. Plant height not only affects crop yield, but is also closely related to crop resistance to abiotic and biotic stresses, especially for dwarf crops. Dwarfing plants can improve plant shape, increase planting density, enhance the photosynthetic efficiency of plant populations, reduce pruning requirements, decrease water consumption, resist lodging, and enhance the transport of crop nutrients to reproductive organs (Wang et al., 2020). Therefore, dwarfism is one of the most important goals in plant breeding. Dwarf breeding is an important strategy for crop breeding such as rice and wheat (Huang et al., 2022; Xu et al., 2022). Since the 1960s, planting semi dwarf varieties had greatly increased crop yields, and dwarf breeding had played a crucial role in increasing crop yields, triggering the first “Green Revolution” (Sasaki et al., 2002; Huang et al., 2021).

Bananas (*Musa* spp.) are perennial monocotyledonous herbaceous plants which belong to the *Musaceae* family. It is not only the most widely consumed fruit known for their nutrition and taste in many tropical and subtropical countries around the world, but also a food crop for millions of people worldwide which is crucial for global food security (De et al., 2008). Compared with some other crops, banana plants grow relatively quickly, have a heavy canopy and shallow roots, and their wind resistance is poor. Therefore, banana plants are prone to serious damage and collapse when encountering typhoons or tropical storms, the false stems of cultivated bananas are easily broken in typhoon-frequented areas, which can cause damage to the growth and yield to a certain extent, and caused enormous economic losses in the banana industry (Takayuki, 2022). In addition, the higher banana plants will consume a large amount of water and nutrients during their growth process. Dwarf banana varieties have a shorter growth period due to their relatively short and sturdy tree shape. The short and strong banana plants of dwarf varieties not only improve wind resistance, but also facilitate cultivation and management (Chen et al., 2014).They are planted in spring and harvested in early winter in general, which effectively avoiding cold damage for banana plant. Due to their short stature, they have a lower chance of being hit by typhoons and have a lower degree of damage of banana. Due to their short tree shape, they can effectively reduce agricultural management costs, promote cultivation and management, shorten the growth cycle and effectively improve the banana yield and economic benefits per mu (Cai et al., 2023; Chen et al., 2014). Therefore, banana dwarfing breeding is an important research direction for cultivating high-quality and high-yield banana varieties, and a better understanding the mechanism of banana dwarfism may help to develop strategies to improve banana dwarfing breeding which is of great significance for the long-term development of the banana industry.

In recent years, with the development of modern high-throughput technologies such as transcriptomics and metabolomics, more and more plants have been studied through this methods to explore key molecules expressed under specific conditions. The latest developments in omics technologies may provide a promising method for revealing the complexity of plant height growth. Chen et al. identified 36 candidate GA metabolic genes between Williams banana 8818 and its dwarf mutant ‘8818-1’ through whole genome screening, and believed that all of these genes may play important roles in banana development, but each gene may play a different role in different tissues or developmental stages (Chen et al., 2016). Deng et al. studied the molecular changes of the semi dwarf banana mutant Aifen No.1 pseudostem compared to its wild-type dwarf cultivation using transcriptomics and metabolomics methods, indicating that gibberellins and related pathways, as well as cell elongation and cell wall modification, may be developing the observed semi dwarf pseudostem phenotype (Deng et al., 2021). Cai et al. identified genes related to plant height through comparative phenotype and transcriptome data of the variety ‘Brazilian Banana’ (‘BX ‘) and its dwarf mutant (‘RK’), suggesting that GA signaling may play a key role in banana plant height development (Cai et al., 2023). Serrano-Misrata et al. believe that key pathways associated with plant height are gibberellin metabolism and signal transduction, cell wall elasticity of swollen cells, and the interactions between GA and other hormone metabolism and signal transduction pathways (Serrano-Mislata and Sablowski, 2018). Deng et al. believe that GA and IAA have a significant impact on banana dwarfism, with GA2ox being a key gene in GA biosynthesis and TDC and YUCCA being key genes in IAA synthesis (Deng et al., 2022). The differential expression of these genes may be the main factor affecting hormone levels and plant height. Although banana genome sequencing was completed in 2012 (D’Hont et al., 2012), there is limited information on controlling plant height in bananas, and current the research on key genes and regulatory mechanisms that control banana plant height is also limited. It is necessary to further study the key genes and their regulatory mechanisms that control plant height during banana development. Dwarf plants are useful materials for exploring and studying dwarf related genes. In this study, we aimed at identifying the differentially expressed genes and metabolites related to plant height through transcriptome sequencing analysis, metabolome sequencing analysis, combined with phenotype indicators and hormone measurement analysis between the widely planted varieties Brazilian banana and local dwarf banana (Df19).The comprehensive analysis of differentially expressed genes and metabolites will help us understand the relevant regulatory mechanisms that control banana plant height and will provide effective theoretical basis for further exploring the dwarfing mechanism of dwarf bananas.

## Materials and methods

### Plant materials and culture conditions

The commercial banana variety Brazilian (*Musa* spp. AAA group, tall plant height phenotype) and local dwarf banana variety ‘Df19’ were selected as materials. Compared to Brazilian banana plants, Df19 is stronger, has shorter fruits, and exhibits dwarfing characteristics. The materials selected in the experiment were planted in the same banana plantation in the suburbs of Danzhou City, Hainan Province, China (E109°C29’ 12 “, N19°C30’ 22”and 136 m above sea level). The area belongs to a tropical marine monsoon climate, with an average annual temperature of 23.5°C, extreme high temperature of 37°C, extreme low temperature of 8 °C, frost free period of 365 days, average annual sunshine hours of 2072 hours, average annual rainfall of 1823mm, and average annual evaporation of 1628 millimeters. The soil is mainly composed of red soil, with a pH value of 5.67. The organic matter content of the surface soil (0-20 cm) in the experimental area is 115.62 g/kg^-1^, total nitrogen content of 0.61 g/kg^-1^, and available phosphorus content. The content is 9.31 mg/kg^-1^ and the effective potassium content is 102.59 mg/kg^-1^. The experiment was conducted under the same growth environment and cultivation mode, using the same fertilizer and water management standards. Each banana resource has three biological replicates. During the sampling and measurement process, the growth period of all these plants is basically the same. We select the second to last banana leaf from the top as the material for the next step of testing and analysis. All samples were frozen in liquid nitrogen and stored at -80°C for future use. All evaluations were conducted using three biological replicates.

### Plant architecture measurement

The banana plants pseudostem height were measured using a steel ruler and the pseudostem circumference were measured using a tape measure in the harvesting period. At the same time, the whole growth period and the fruit weight in the harvesting period were also measured.

### Plant hormone assays

The content of ABA, GA3, IAA, JA and BR were determined using an enzyme linked immunosorbent assay kit according to the manufacturer’s instructions. The reagent kit was purchased from Jiancheng Industrial Co., Ltd (Nanjing, China). The experiment was conducted according to the manufacturer’s instructions. Grind the main leaves sample (0.5g) into powder in liquid nitrogen and extract with phosphate buffer (pH 7.0). Each treatment processing includes three repetitions.

### RNA extraction, library preparation, and Illumina NovaSeq

The banana plant total RNA was extracted using the RNAprep Pure Plant Kit (Tiangen, Beijing, China) according the instructions provided by the manufacturer. Sample from the second to last leaf at the top, tear the leaf in half along the vein, mix and sample for RNA extraction. Total RNA concentration and purity was measured using NanoDrop 2000 (Thermo Fisher Scientific, Wilmington, DE). Total RNA integrity was assessed using the RNA Nano 6000 Assay Kit of the Agilent Bioanalyzer 2100 system (Agilent Technologies, CA, USA). A total amount of 1μg RNA per sample was used as input material for the RNA sample preparations. Sequencing libraries were generated using Hieff NGS Ultima Dual-mode mRNA Library Prep Kit for Illumina (Yeasen Biotechnology (Shanghai) Co., Ltd.) following manufacturer’s recommendations and index codes were added to attribute sequences to each sample. RNA-seq libraries were produced using Illumina NovaSeq6000 sequencing performed by the Biomarker Biotechnology Corporation (Beijing, China).

### Sequencing data analysis and real-time qRT-PCR

Raw data (raw reads) of fastq format were firstly processed through in-house perl scripts. In this step, clean data(clean reads) were obtained by removing reads containing adapter, reads containing ploy-N and low quality reads from raw data. At the same time, Q20, Q30, GC content and sequence duplication level of the clean data were calculated. All the downstream analyses were based on clean data with high quality. The adaptor sequences and low-quality sequence reads were removed from the data sets. Raw sequences were transformed into clean reads after data processing. These clean reads were then mapped to the reference genome sequence (Musa_acuminata.DH_Pahang_v4.genome.fa) using HISAT2 software. The StringTie Reference Annotation Based Transcript (RABT) assembly method was used to construct and identify both known and novel transcripts from HISAT2 alignment results. Differential expression analysis of two conditions/groups was performed using the DESeq2. DESeq2 provide statistical routines for determining differential expression in digital gene expression data using a model based on the negative binomial distribution. The resulting P values were adjusted using the Benjamini and Hochberg’s approach for controlling the false discovery rate. Genes with an adjusted P-value <0.01 & Fold Change≥2 found by DESeq2 were assigned as differentially expressed. Gene function was annotated based on the following databases: Nr (NCBI non-redundant protein sequences); Pfam (Protein family); KOG/COG (Clusters of Orthologous Groups of proteins); Swiss-Prot (A manually annotated and reviewed protein sequence database); KO (KEGG Ortholog database); GO (Gene Ontology). To verify the transcriptome results, the real-time quantitative PCR (RT-qPCR) analysis using the gene-specific primers listed in **Supplementary Table S1**. Three independent biological replicates were used for each sample. The UBQ gene was used as an internal control to standardize the expression results (Chen et al., 2011). The relative expression levels were determined by 2^−ΔΔCt^ methods (Livak and Schmittgen, 2001). The data were subjected to ANOVA with Duncan’s multiple range test at P < 0.05. Each reaction was performed in triplicates.

### Metabolite extraction and LC-MS/MS analysis

A widely targeted metabolome was obtained from Biomarker Biotechnology Corporation (Beijing, China). The LC/MS system for metabolomics analysis is composed of Waters Acquity I-Class PLUS ultra-high performance liquid tandem Waters Xevo G2-XS QTof high resolution mass spectrometer and the injection volume were 1μL. Waters Xevo G2-XS QTOF high resolution mass spectrometer can collect primary and secondary mass spectrometry data in MSe mode under the control of the acquisition software (MassLynx V4.2, Waters). In each data acquisition cycle, dual-channel data acquisition can be performed on both low collision energy and high collision energy at the same time. The low collision energy is 2V, the high collision energy range is 10~40V, and the scanning frequency is 0.2 seconds for a mass spectrum. The parameters of the ESI ion source are as follows: Capillary voltage: 2000V (positive ion mode) or -1500V (negative ion mode); cone voltage: 30V; ion source temperature: 150°C; desolvent gas temperature 500°C; backflush gas flow rate: 50L/h; Desolventizing gas flow rate: 800L/h. The raw data collected using MassLynx V4.2 is processed by Progenesis QI software for peak extraction, peak alignment and other data processing operations, based on the Progenesis QI software online METLIN database and Biomark’s self-built library for identification, and at the same time, theoretical fragment identification and mass deviation all are within 100ppm. After normalizing the original peak area information with the total peak area, the follow-up analysis was performed. Principal component analysis and Spearman correlation analysis were used to judge the repeatability of the samples within group and the quanlity control samples. The identified compounds are searched for classification and pathway information in KEGG, HMDB and lipidmaps databases. According to the grouping information, calculate and compare the difference multiples, T test was used to calculate the difference significance pvalue of each compound. The R language package ropls was used to perform OPLS-DA modeling, and 200 times permutation tests was performed to verify the reliability of the model. The VIP value of the model was calculated using multiple cross-validation. The method of combining the difference multiple, the P value and the VIP value of the OPLS-DA model was adopted to screen the differential metabolites. The screening criteria are FC>1, PP value<0.05 and VIP>1. The difference metabolites of KEGG pathway enrichment significance were calculated using hypergeometric distribution test.

### Combined transcriptome and metabolome analyses

In addition to individual analyses for transcriptome sequencing and metabolome profiling, we performed co-joint analyses on the differentially expressed genes (DEGs) and differentially accumulated metabolites (DAMs) to determine the degree of enrichment of pathways. The Corson program in R was used to calculate the PCC of genes and metabolites and a correlation coefficient cluster heatmap of DEGs and DAMs was drawn. Gene-metabolite networks with a Pearson correlation coefficient (PCC) > 0.8 were used to construct the transcriptmetabolite network.

## Results

### Comparative analysis of phenotypic characteristics

**Figure 1:**
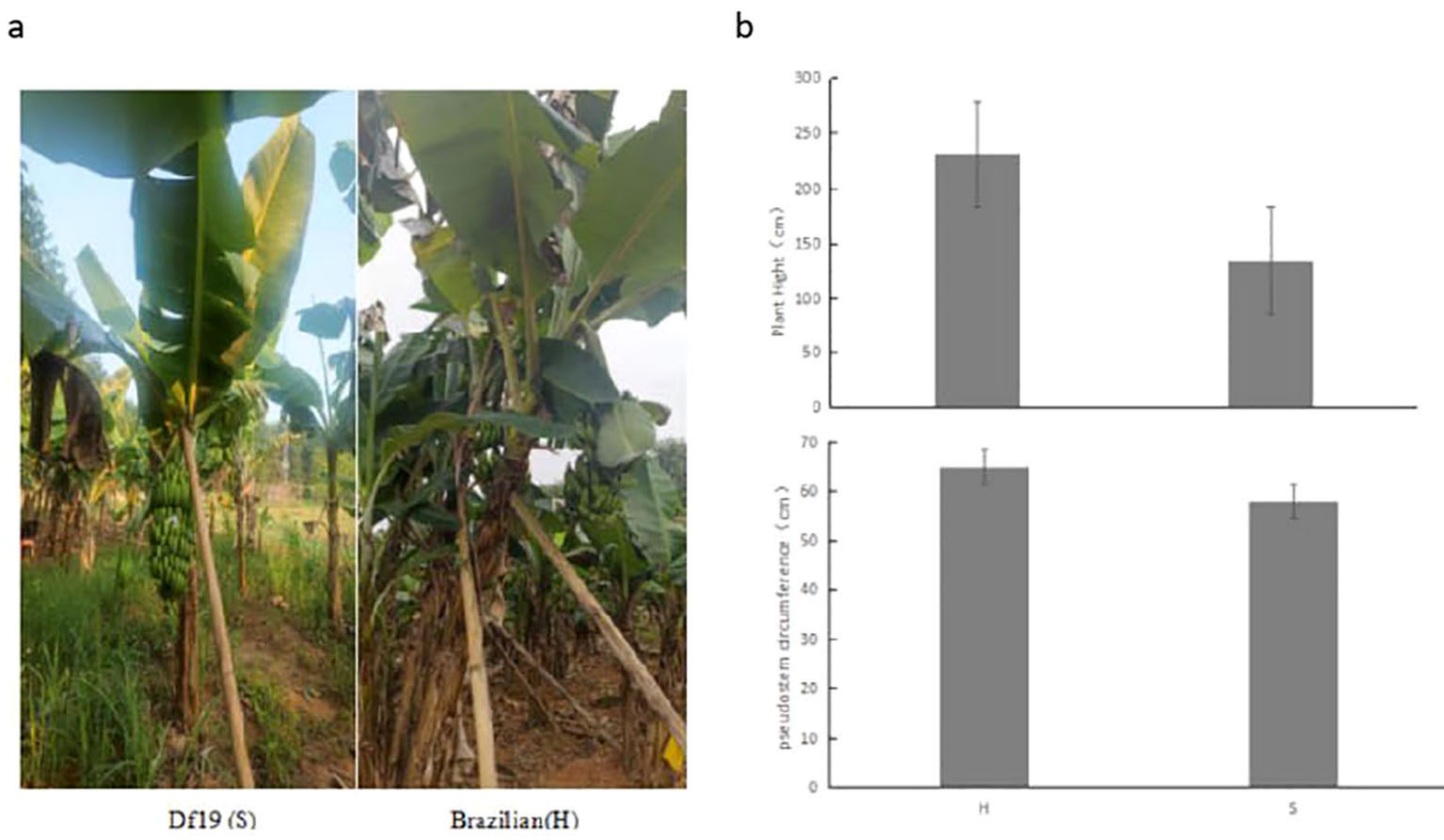
**(A)** Phenotype of Df19 (S) and Brazilian (H) in the field and **(B)** comparison of plant height and pseudostem circumference between Df19 (S) and Brazilian (H).

Based on observations and measurement results, we have found that the plant height of adult Df19 banana plants (S) planted is between 1.2-1.5 meters and the pseudostem circumference is between 0.56-0.61 meters, while the plant height of Brazilian banana (H) plants is between 2.2-2.5 meters and the circumference of the pseudostem is between 0.61-0.69 meters in the experimental field. The whole growth period of Df19 is 280-295 days, while that of Brazilian banana is 320-360 days. In addition, the fruit weight of Df19 bananas during the harvest period is 10-14 kg, while the fruit weight of Brazilian banana plants is 21-28 kg (**Figure 1**).

### Differences in the ABA, GA3, IAA, JA and BR content

In the present study, we studied the differences in ABA, GA3, IAA, JA and BR between the two banana varieties. The results showed that the ABA of the Brazilian (H) was 10.79% higher than that of Df19 (S). The GA3 of Brazilian (H) was 19.59% higher than that of Df19 (S). The IAA of Brazilian (H) is 0.4% higher than that of Df19 (S). The JA of Brazilian (H) is 10.09% higher than that of Df19 (S). The BR of Df19 (S) was 24.91% higher than that of Brazilian (H).

### RNA sequencing and data analysis

To analyze the gene expression differences between Df19 (S) and Brazilian (H) plants, the second leaves (number from the shoot tip) of each varieties were selected as experimental materials, which were labeled as H and S respectively. With three biological replicates, we performed Illumina 6000 sequencing for each RNA sample (H1, H2, H3, S1, S2 and S3) and generated six sub transcriptomes. The Spearman correlation coefficient graphs between different sample pairs clearly indicate that each sample was reliable with a good reproducibility (**Supplementary Table S2**). After removing low-quality reads, 63,151,401 (H) and 62,784,800 (S) clean reads generated a total of 37.70 Gb of clean data, with Q20 values>97.1% and Q30 values>92.4%, and the overall GC content was more than 50% (**Table 1**), indicating that transcriptome sequencing data can be used for further analysis. Based on the comparison between sequencing readings and reference genomes, over 89.25% of mapping readings can be found in all libraries (**Supplementary Table S3**). The results indicate that the selection of the reference genome is appropriate and the sequencing results are acceptable. Therefore, they are suitable for subsequent analysis. Quantification of gene expression results for Df19 (S) and Brazilian (H) samples is shown in **Supplementary Figure S1A** and when the Fragments Per Kilobase of transcript per Million fragments mapped (FPKM) >1 was used as a threshold for determining gene expression, the FPKM of Brazilian (H) banana samples was slightly higher than that of Df19 (S) banana samples. We used Pearson correlation coefficient r as an evaluation index for biological repeat correlation, and the closer the calculated r2 is to 1, the stronger the correlation between two repeat samples. The Pearson correlation coefficient between Brazilian (H) and Df19 (S) replicates is ranged from 0.765 to 0.9980 which indicating that the quantitative results of gene expression are highly reliable (**Supplementary Figure S1B**).

**Table 1:** Statistics of 6 samples for banana transcriptome.

Samples	Total Read	Clean Read	Clean Base	Q20	Q30	GC
H-1	42,566,738	21283369	6.38G	97.43	92.96	50.46
H-2	41,148,788	20574394	6.16G	97.57	93.39	50.32
H-3	42,587,276	21293638	6.38G	97.15	92.44	50.67
S-1	41,338,466	20669233	6.19G	97.67	93.7	50.9
S-2	42,292,816	21146408	6.33G	97.45	93.14	50.75
S-3	41,938,318	20969159	6.28G	97.28	92.8	50.67

### Analysis of differentially expressed genes

**Figure 2:**
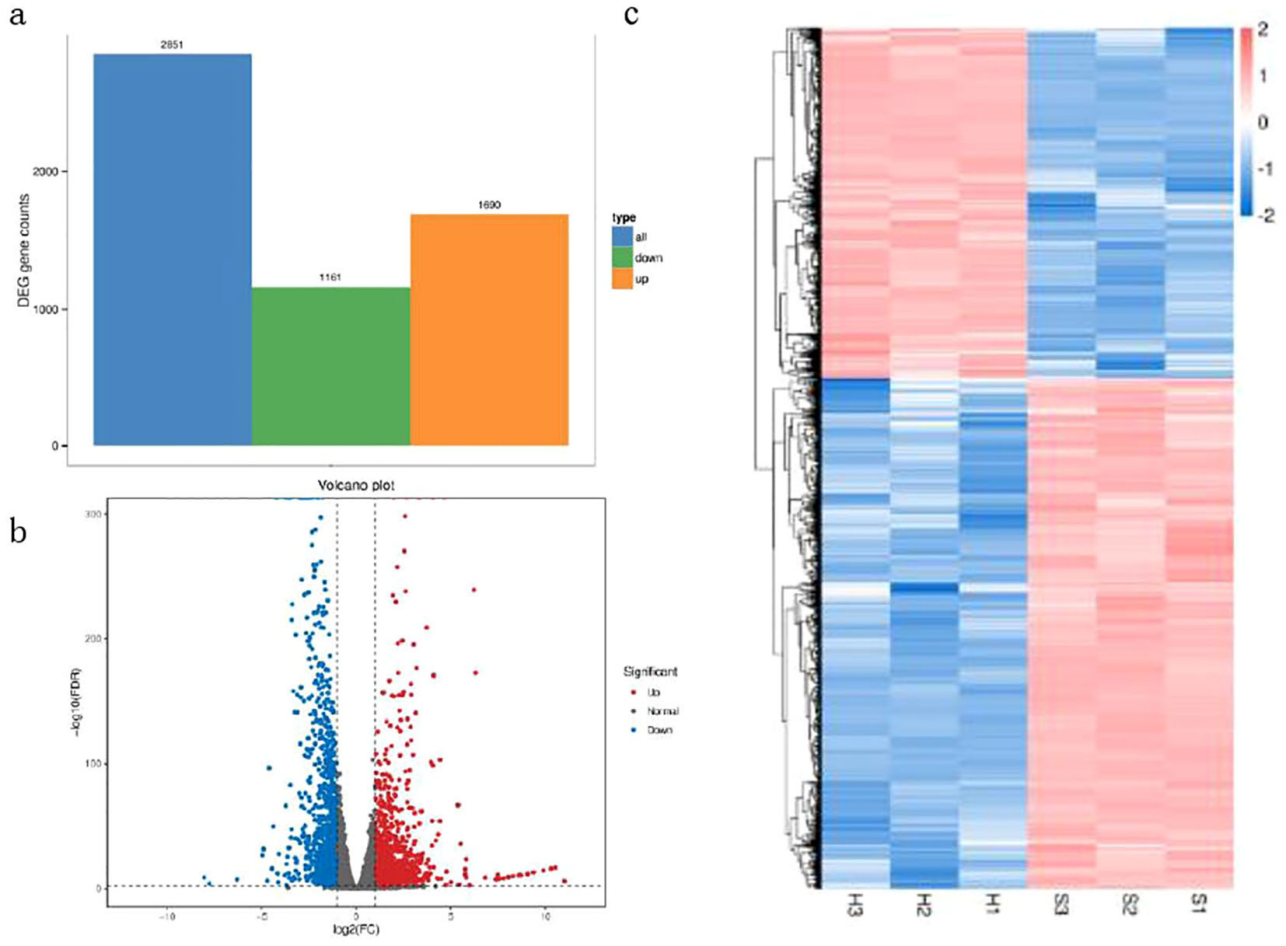
Analysis of transcriptome sequencing data for leaves of banana between H vs S. **(A)** Number of differentially expressed genes (DEGs). **(B)** Statistics for differentially expressed genes (DEGs). **(C)** Cluster diagram of differentially expressed genes (DEGs).

Differential expression analysis was conducted using DESeq2 which provides a statistical routine for determining differential expression (Anders and Huber, 2010). FDR (Error Detection Rate) control method was used to identify the threshold of P-values and calculate the significance of differences. DEGs were identified between Brazilian (H) and Df 19 (S) with the screening thresholds for selection of DEGs was FDR (q value)<0.01 and | log2 (fold change) | ≥ 2. Therefore, a total of 2851 DEGs were identified between Brazilian (H) and Df19 (S). Among these DEGs, there were 1690 up-regulated genes (59.3%) and 1161 down-regulated genes (40.7%) (**Figure 2A**). In order to confirm the validity of the RNA-Seq based transcript abundance of genes, a qRT-PCR analysis of four DEGs was performed. The UBQ genes were used as internal controls. The results showed that the expression patterns of the four DEGs obtained through qRT-PCR had consistent trend with the results of the transcriptome sequencing and validates the reliability of the Illumina sequencing results (**Supplementary Figure S3**). The gene expression levels between Brazilian (H) and Df19 (S) are shown in **Figure 2B** according to the value of expression abundance (RPKM). The gene expression cluster analysis based the heatmap of between Brazilian (H) and Df19 (S) was presented in **Figure 2C**. Top-5 highly down-regulated genes in Df19 banana were heavy metal-associated isoprenylated plant protein (Macma4_03_g11070), hevamine-A-like (Macma4_08_g34270), protein of unknown function (NewGene_2743), heat shock 70 kDa protein-like (Macma4_05_g04610) and ethylene-responsive transcription factor ERF109-like (Macma4_05_g05690).Top-5 genes that showed higher log2FoldChange values (higher expression in Df19) were protein TRACHEARY ELEMENT DIFFERENTIATION-RELATED 7A-like (Macma4_05_g29280), protein TRACHEARY ELEMENT DIFFERENTIATION-RELATED 7A-like (Macma4_05_g29290), mannan endo-1,4-beta-mannosidase 6-like (Macma4_03_g09790), GDSL esterase/lipase At2g42990-like (Macma4_02_g08730), and glycosyl hydrolase 5 family protein-like (Macma4_06_g23170). The changes in gene expression indicate that pseudostem height of banana plants may be influenced by factors such as ethylene, hevamine, glycosyl hydrolase, esterase/lipase, endo-1,4-beta-mannosidase.

### GO enrichment analysis of DEGs

**Figure 3:**
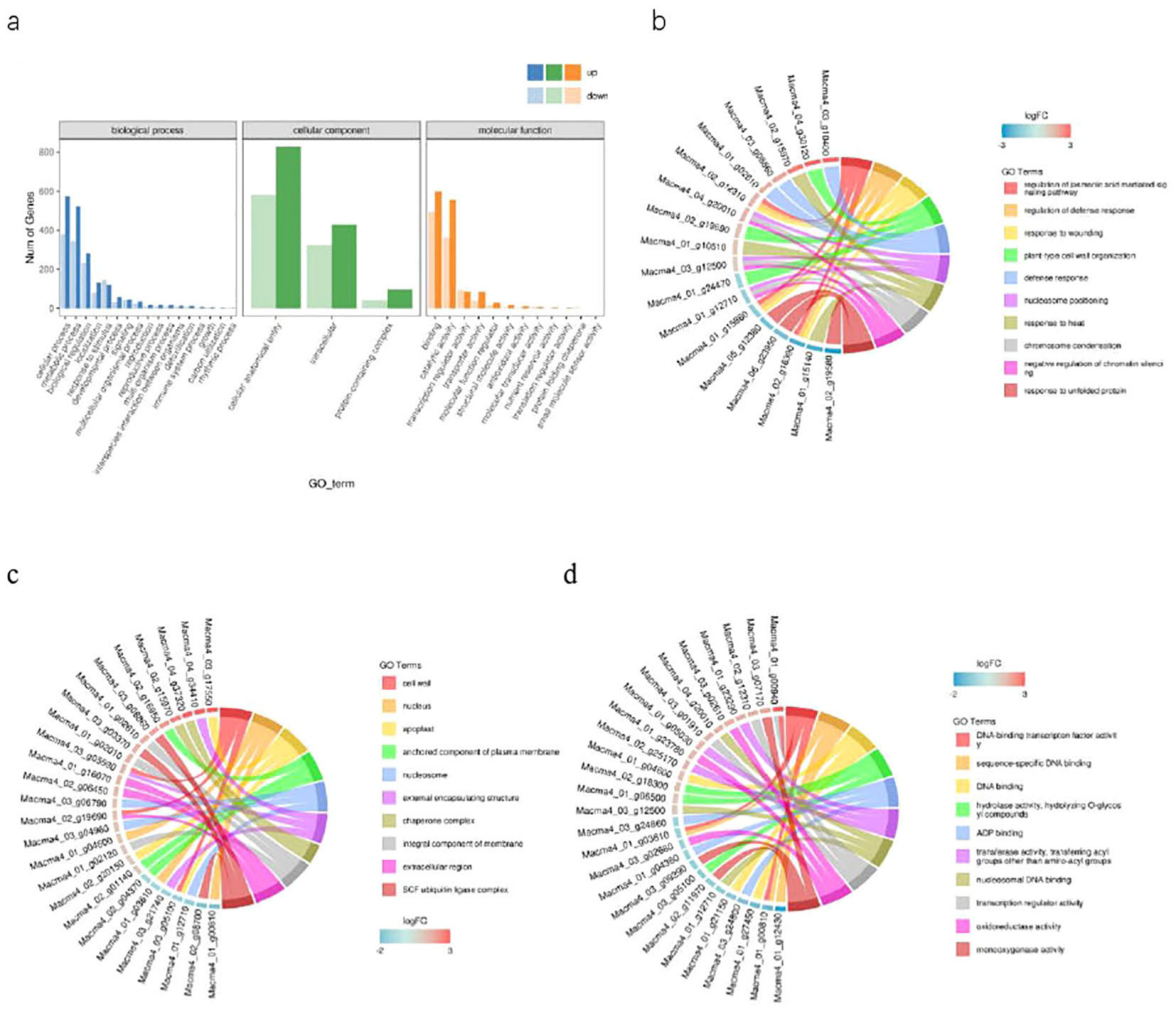
GO classification enrichment analysis of DEGs between H vs S. **(A)** Statistical chart of GO annotation classification. **(B)** The top ten GO enrichment terms in the biological process. **(C)** The top ten GO enrichment terms in the cellular component. **(D)** The top ten GO enrichment terms in the molecular function.

We used the GO analysis to classify the functions of transcriptional sequences with the threshold of p < 0.01. These DEGs were divided into 41 groups of three major categories with biological process, cellular component and molecular function between Brazilian (H) and Df19 (S) comparison. Among them, 40.33% were belong to the “biological process category”, 29.05% were belong to the “cellular component category”, and 30.62% were belong to the “molecular function category”. In the comparison of GO classification, the main classifications of biological processes were “metabolic process”, “cellular process”, “biological regulation”, “localization” and “response to stimulus”. The main classifications of cellular components were “intracellular”, “protein-containing complex” and “cellular anatomical entity”. The main classifications of molecular function were “binding”,”catalytic activity”, “transcription regulator activity” and “transporter activity” (**Figure 3A**). The top ten GO enrichment terms in the biological process were “regulation of jasmonic acid mediated signaling pathway (GO:2000022)”, “regulation of defense response (GO:0031347)”, “response to wounding (GO:0009611)”, “plant-type cell wall organization (GO:0009664)”, “defense response (GO:0006952)”, “nucleosome positioning (GO:0016584)”, “response to heat (GO:0009408)”, “chromosome condensation (GO:0030261)”, “negative regulation of chromatin silencing (GO:0031936)” and “response to unfolded protein (GO:0006986)” (**Figure 3B**). The top ten GO enrichment terms in the cellular component were “cell wall (GO:0005618)”, “nucleus (GO:0005634)”, “apoplast (GO:0048046)”, “anchored component of plasma membrane (GO:0046658)”, “nucleosome (GO:0000786)”, “external encapsulating structure (GO:0030312)”, “chaperone complex (GO:0101031)”, “integral component of membrane (GO:0016021)”, “extracellular region (GO:0005576)” and “SCF ubiquitin ligase complex (GO:0019005)” (**Figure 3C**). The top ten GO enrichment terms in the molecular function were “DNA-binding transcription factor activity (GO:0003700)”,”sequence-specific DNA binding (GO:0043565)”, “DNA binding (GO:0003677)”, “hydrolase activity, hydrolyzing O-glycosyl compounds (GO:0004553), “ADP binding (GO:0043531)”, “transferase activity, transferring acyl groups other than amino-acyl groups (GO:0016747)”, “nucleosomal DNA binding (GO:0031492)”, “transcription regulator activity (GO:0140110)”, “oxidoreductase activity (GO:0016491)” and “monooxygenase activity (GO:0004497)” (**Figure 3D**). Gene Ontology (GO) enrichment analyses revealed that “metabolic process”, “cellular process” and “biological regulation”-related genes showed significant differences in expression levels. This result suggested that these DEGs might occupy an important position in the molecular mechanism of height of banana plant. The differential expressions of JA-related genes may be one of the main reasons for causing the difference in plant height between Brazilian (H) and Df19 (S).

### KEGG pathway analysis of DEGs

**Figure 4:**
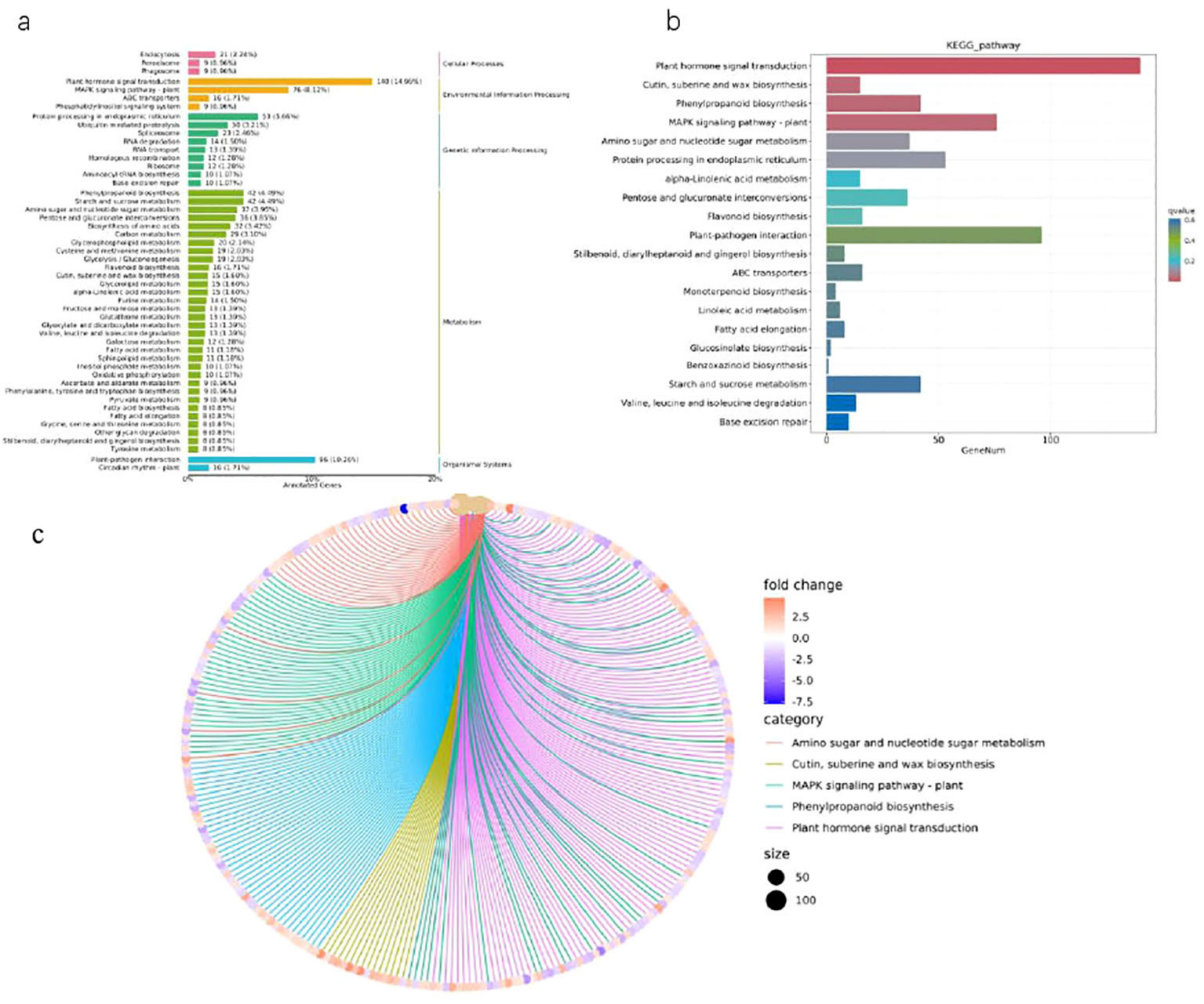
KEGG enrichment analysis of DEGs between H vs S. **(A)** KEGG classification map of differentially expressed genes. **(B)** KEGG enrichment bar chart of differentially expressed genes. **(C)** KEGG enrichment network diagram of differentially expressed genes.

In order to comprehensively understand the active biological pathways of DEG between Brazilian (H) and Df19 (S), the affected biochemical pathways were analyzed based on expression profiles. The results showed that 1364 DEGs were annotated into 127 metabolic pathways. Among them, 545 up-regulated DEGs genes were annotated into 116 metabolic pathways, and 391 down-regulated DEG genes were annotated into 88 metabolic pathways. Among 127 metabolic pathways, 37 pathways were annotated as up-regulated DEG, 9 pathways were annotated as down-regulated DEG, and 80 pathways were annotated as both up-regulated and down-regulated genes. The 127 KEGG pathways include five main KEGG categories, including cellular processes, environmental information processing, genetic information processing, metabolism, and biological systems (**Supplementary Table S4**, **Figure 4A**). The DEGs were made additional distinctions based on its different biological functions. These DEGs genes mainly belonged to KEGG pathways, including plant hormone signal transduction, cutin, suberine and wax biosynthesis, phenylpropanoid biosynthesis, MAPK signaling pathway-plant, amino sugar and nucleotide sugar metabolism, protein processing in endoplasmic reticulum, alpha-Linolenic acid metabolism, pentose and glucuronate interconversions, flavonoid biosynthesis, plant-pathogen interaction, stilbenoid, diarylheptanoid and gingerol biosynthesis, ABC transporters, monoterpenoid biosynthesis, linoleic acid metabolism, fatty acid elongation, glucosinolate biosynthesis, benzoxazinoid biosynthesis, starch and sucrose metabolism. Among the pathways significantly enriched in DEGs, plant hormone signal transduction (140 members in bananas) was the largest complex. It followed by plant-pathogen interaction (96 members in banana), MAPK signaling pathway-plants (76 members in banana), protein processing in endoplasmic reticulum (53 members in banana), phenylpropanoid biosynthesis (42 members in banana), starch and sucrose metabolism (42 members in banana) and so on (**Table 2**, **Figure 4B**). Plant hormone signal transduction is a kind of metabolic pathway with the maximum number of genes play a role in the process of plant development. In this study, there were 16 DEGs with KEGG annotation in flavonoid biosynthesis pathway. Furthermore, The top five KEGG enrichment terms (qvalue) were cutin, suberine and wax biosynthesis, Phenylpropanoid biosynthesis, MAPK signaling pathway-plants, amino sugar and nucleotide sugar metabolism and protein processing in endoplasmic reticulum (**Figure 4C**).

**Table 2:** KEGG pathways of DEGs.

#	ID	Pathway	Annotation	p-Value
1	ko04075	Plant hormone signal transduction	140	6.78E-09
2	ko00073	Cutin, suberine and wax biosynthesis	15	0.000740106
3	ko00940	Phenylpropanoid biosynthesis	42	0.001049249
4	ko04016	MAPK signaling pathway - plant	76	0.001163383
5	ko00520	Amino sugar and nucleotide sugar metabolism	37	0.004969987
6	ko04141	Protein processing in endoplasmic reticulum	53	0.006124687
7	ko00592	alpha-Linolenic acid metabolism	15	0.012749974
8	ko00040	Pentose and glucuronate interconversions	36	0.01592536
9	ko00941	Flavonoid biosynthesis	16	0.018777596
10	ko04626	Plant-pathogen interaction	96	0.039810393
11	ko00945	Stilbenoid, diarylheptanoid and gingerol biosynthesis	8	0.047659422
12	ko02010	ABC transporters	16	0.054430958
13	ko00902	Monoterpenoid biosynthesis	4	0.059700027
14	ko00591	Linoleic acid metabolism	6	0.063363849
15	ko00062	Fatty acid elongation	8	0.07529239
16	ko00966	Glucosinolate biosynthesis	2	0.07700724
17	ko00402	Benzoxazinoid biosynthesis	1	0.079863481
18	ko00500	Starch and sucrose metabolism	42	0.087030116

### DEGs involved in plant hormone signal transduction pathways

**Figure 5:**
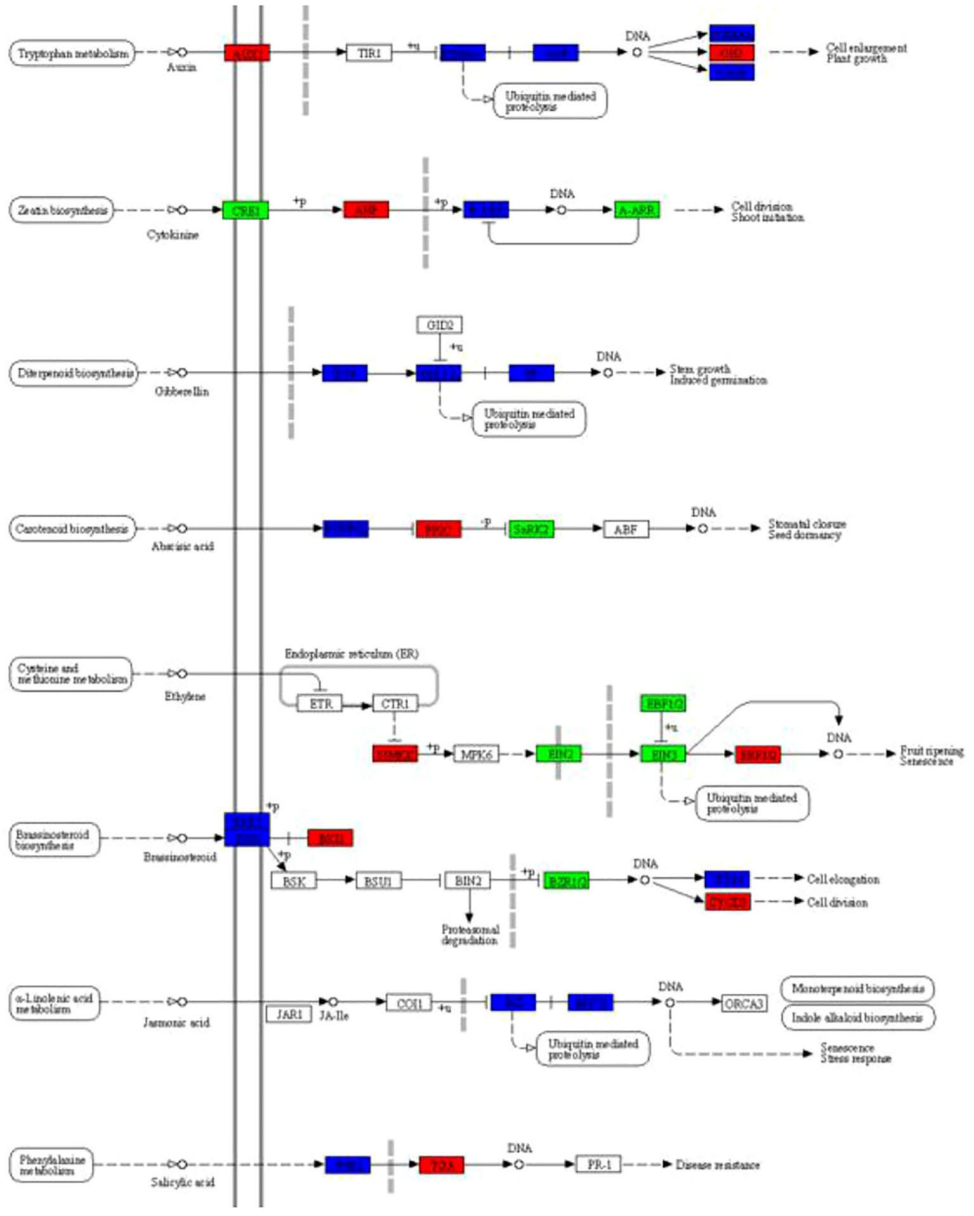
Effects of plant hormone signal transduction between H vs S. Up-regulated genes marked in red box, down-regulated genes marked in green box, and both up-regulated and down-regulated marked in blue box, white represents no significant change.

Based on the above KEGG enrichment analysis, we further analyzed the DEGs related to the plant hormone signal transduction pathway between Brazilian (H) and Df19 (S) including auxin, cytokinin, gibberellin, abscisic acid, ethylene, brassinosteroid, jasmonic acid and salicylic acid (**Figure 5**). Auxin is a small organic acid that affects cell division, elongation, and differentiation in higher plants, and has a significant impact on the final shape and function of all cells and tissues in higher plants (Ljung, 2013). The Aux/IAA family plays a crucial role in inhibiting gene expression levels activated by auxin response factors (ARFs) (Luo et al., 2018). The GH3 acylamide synthase family plays a crucial role in the binding of IAA to acyl-containing small molecule substrates (such as amino acids and jasmonic acid) that regulates plant growth and stresses by regulating auxin homeostasis (Luo et al., 2023). From **Figure 5**, it can be seen that the expression of auxin influx carrier (AUX1 LAX family) (Macma4_08_g07310) and auxin responsive GH3 gene family (Macma4_01_g04590) in the tryptophan metabolism pathway were up-regulated between Brazilian (H) vs Df19 (S) which is consistent with the decrease of IAA content measured in DF19 bananas, indicating that the expression changes of these key genes involved in the auxin signaling pathway are a component of reduced auxin content in dwarf plants. Cytokinins (CK) are involved in almost all kinds of plant growth and development. The signal of cytokinin (CK) is mainly mediated by histidine kinase (AHK), histidine phosphotransferase protein (AHP), and response regulatory factor (ARR). Among them, CRE1(AHK4) acts as cytokinin receptors and B-ARR acts as response regulatory factor in the CK signaling pathway (Veerabagu et al., 2012; Suzuki et al., 2000). Between Brazilian (H) vs Df19 (S), the expression of AHP (Macma4_05_g31020) was up-regulated while the expression of CRE1 (Macma4_08_g24290) and B-ARR (Macma4_06_g20190; Macma4_08_g30720) were down-regulated, which may be regulate the growth of banana plant. The phytohormone gibberellin (GA) plays a key role in promoting stem elongation in plants (Zhou et al., 2016). DELLA protein act as growth repressors plays a negative regulatory role in gibberellin (GA) signaling transduction (Sun, 2011). PIF4 is phytochrome-interacting factor 4 and GIBBERELLIN-INSENSITIVE DWARF1 (GID1) is Gibberellin receptor which inhibit the repression activity of DELLAs on GA signaling (Griffiths et al., 2006). In our study, it was revealed that the expression of four GID1 (Macma4_06_g17910; Macma4_08_g05590; Macma4_09_g08040; Macma4_11_g06090) were up-regulated and one GID1 (Macma4_06_g32180) was down-regulated, the expression of seven DELLAs (Macma4_04_g03710; Macma4_04_g32170; Macma4_06_g31760; Macma4_06_g38180; Macma4_07_g15210; Macma4_11_g02690; Macma4_11_g05790)were up-regulated and eight DELLAs (Macma4_03_g27910; Macma4_04_g07170; Macma4_06_g26910; Macma4_06_g37760; Macma4_07_g29950; Macma4_08_g03450; Macma4_09_g12390; Macma4_09_g26660) was down-regulated, the expression of four PIF4s (Macma4_09_g15580; Macma4_01_g10660; Macma4_03_g06570; Macma4_04_g40630) were up-regulated and one PIF4s (Macma4_06_g34670) was down-regulated between Brazilian (H) vs Df19 (S). Abscisic acid (ABA) plays an important role in regulating various physiological processes in plants, especially stress physiological processes (Park et al., 2009). PP2C (protein phosphatase 2C) as a key negative regulatory factor, plays an important regulatory role in the activation of ABA signaling. SNRK2 is serine/threonine-protein kinase and the development of plant organs is mainly controlled by the interaction between SnRK2 and the plant hormone abscisic acid (ABA) (Hasan et al., 2022). In our study, the expression of PP2C (Macma4_11_g23370) were up-regulated and the expression of SNRK2 (K14498 Macma4_03_g01540) was down-regulated between Brazilian (H) vs Df19 (S). Ethylene is an important signaling molecule which involved in various physiological processes. SIMMKK is mitogen-activated protein kinase 4/5 and ERF1/2 is ethylene-responsive transcription factor 1 which belong to ERF(ethylene responsive factor)family. EIN2 and EIN3 are both ethylene-insensitive protein and EBF1/2 is EIN3-binding F-box protein. In our study, the expression of SIMMKK (Macma4_06_g21720; Macma4_09_g13830; Macma4_09_g32860) and ERF1/2 (Macma4_11_g21380) were up-regulated and the expression of EIN2 (Macma4_07_g01320; Macma4_09_g10760), EIN3 (Macma4_08_g22960; Macma4_09_g17840) and EBF1/2 (Macma4_02_g19260; Macma4_04_g39460; Macma4_06_g07040; Macma4_09_g30820; Macma4_10_g30580) were down-regulated between Brazilian (H) vs Df19 (S). Brassinolide, also known as brassinolide, is a natural plant hormone that is widely present in organs such as pollen, seeds, stems, and leaves of plants. BRK1is BRI1 kinase inhibitor 1, CYCD3 is cyclin D3 protein and BZR1/2 is brassinosteroid resistant 1/2 in plant. In our study, the expression of BRK1 (Macma4_04_g08700; Macma4_05_g05650; Macma4_08_g24670) and CYCD3 (Macma4_07_g05610) were up-regulated and the expression of BZR1/2 (Macma4_08_g29280; Macma4_09_g05560) were down-regulated between Brazilian (H) and Df19 (S). Jasmonic acid (JA) participates in the regulation of plant growth and development, as well as stress and defense responses. As jasmonate ZIM domain-containing protein, JASMONATE ZIM-DOMAIN (JAZ) protein is an inhibitor of the JA signaling pathway. In our study, it was revealed that the expression of four JAZ (Macma4_02_g12310; Macma4_10_g06810; Macma4_10_g21960; Macma4_10_g22390) were up-regulated and thirteen JAZ (Macma4_01_g15860; Macma4_02_g25340; Macma4_03_g01960; Macma4_05_g05150; Macma4_05_g25520; Macma4_05_g28320; Macma4_06_g22320; Macma4_06_g34370; Macma4_07_g01260; Macma4_08_g00550; Macma4_08_g19160; Macma4_09_g14550; Macma4_11_g09370) were down-regulated, the expression of five MYC2 transcription factor (Macma4_01_g03860; Macma4_01_g20870; Macma4_02_g12000; Macma4_04_g19090; Macma4_06_g08980) were up-regulated and four MYC2 transcription factor (Macma4_03_g16980; Macma4_03_g28880; Macma4_08_g27870; Macma4_09_g24880) were down-regulated between Brazilian (H) vs Df19 (S). As a structurally simple phenolic plant hormone, salicylic acid plays a role in plant growth and development, photosynthesis, transpiration, as well as ion absorption and transport (An and Mou, 2011). NPR1 is the main core factor of salicylic acid signal transduction, and it interacts with the TGA transcription factor family, possibly serving as a transcriptional co activator of SAR gene expression (Cao et al., 1997). In our study, the expression of transcription factor TGA (Macma4_10_g34180) were up-regulated between Brazilian (H) vs Df19 (S) which may benefit banana plant growth.

### DEGs encoding transcription factors

**Figure 6:**
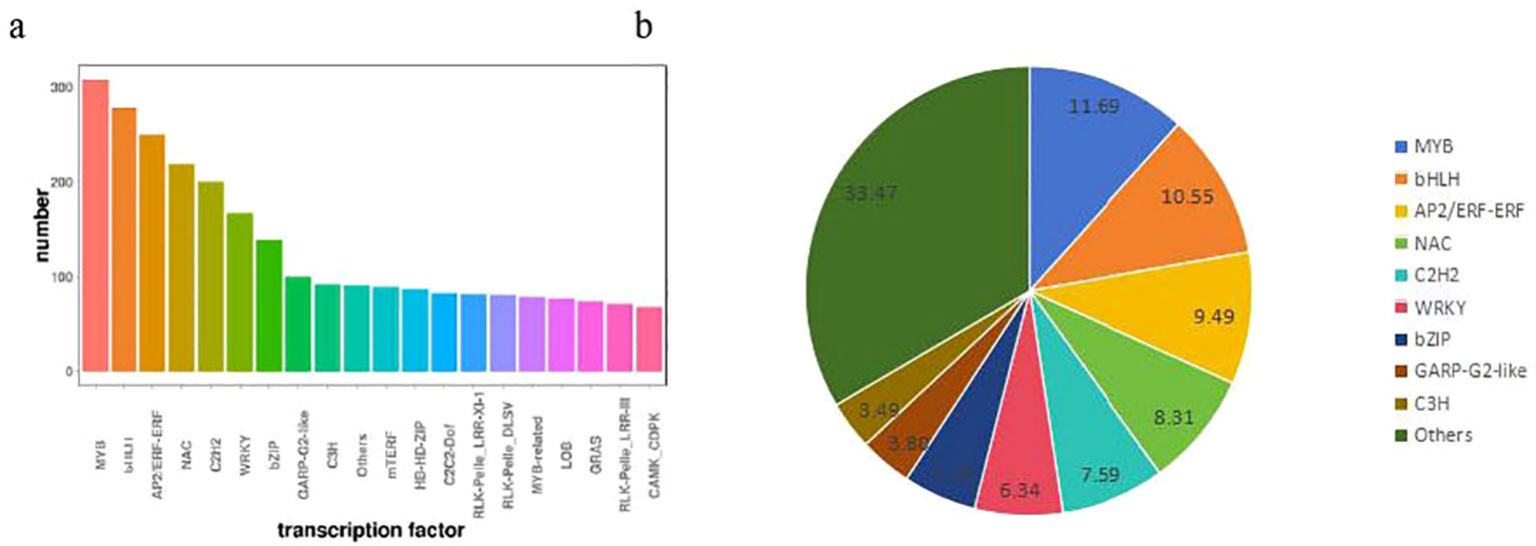
**(A)** The homologue classification and **(B)** the proportion of distribution of transcription factor gene families expressed between H vs S.

As upstream regulatory factors of metabolic pathways, transcription factors play a crucial role in gene expression (Shan et al., 2013). In this study, in order to evaluate the complex signal pathway network in responses, we make further comparation in the expression profiles of the TFs of banana plant (**Supplementary Table S5**). The TFs were separated in to 20 groups according to homologue classification. Most of the TFs belonged to the MYB, bHLH, AP2/ERF-ERF, NAC and other families (**Supplementary Table S5**, **Figure 6A**). In addition, six TF families account for 53.97% of these groups, including MYB (308 numbers), bHLH (278 numbers), AP2/ERF-ERF (250 numbers), NAC (219 numbers), C2H2 (200 numbers) and WRKY (167numbers), played important roles in response to gene expression in banana plant (**Figure 6B**). These transcription factors genes may contribute to study the molecular mechanism of banana plant growth.

### Differentially accumulated metabolites analysis

We also studied the Brazilian (H) and Df19 (S) banana leaves metabolome to understand the role of metabolites. Based on the LC-QTOF platform, metabolomic qualitative and quantitative analysis was conducted on 6 samples, and a total of 14164 peaks were detected and that 3322 metabolites were identified in both groups. The various metabolites were classified into 7 different categories including 456 biosynthesis of other secondary metabolites, 225 amino acid metabolism, 189 lipid metabolism, 182 metabolism of terpenoids and polyketides, 149 metabolism of cofactors and vitamins, 117 carbohydrate metabolism. The analysis of the composition showed the highest content of biosynthesis of other secondary metabolites, accounting for 13.73% of the total metabolites. The principal component analysis (PCA) of the metabolome clearly distinguished between Brazilian (H) vs Df19 (S), each consisting of three biological replicates. The results showed that the cumulative contribution rate of the two main components, PC1 and PC2, reached 67.2% (**Supplementary Figure S3A**), indicating that the experiment is reproducible and reliable. In this study, the OPLS-DA model was used to compare samples and evaluate the differences between Brazilian (H) vs Df19 (S). The result is R2X=0.825, R2Y=1, Q2 = 0.967. The Q2 values of Y and O are 0.967, which is greater than 0.9.The established OPLS-DA model showed that the model is very good and can be used for further analysis (**Supplementary Figure S3B**).

**Figure 7:**
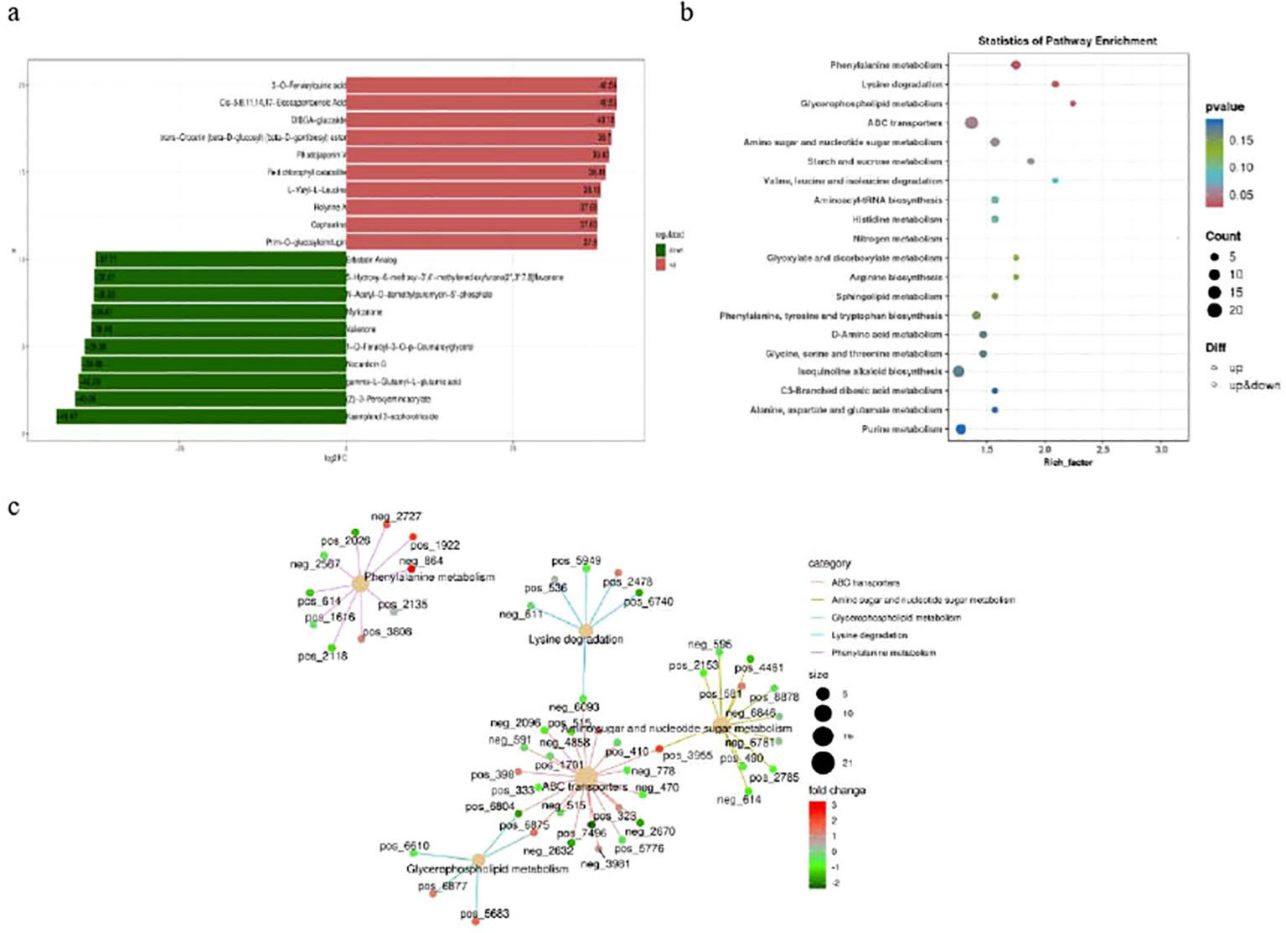
Differential metabolite of banana between H vs S. **(A)** Top-10 up-accumulated (shown in red bars) and top-10 down-accumulated metabolites (shown in green bars). **(B)** Scatter plot of 20 KEGG pathways to which the differentially accumulated metabolites were enriched. **(C)** KEGG enrichment network diagram.

Based on the criteria of a fold change (FC) value ≥ 1, P value ≤ 0.05 and a variable importance in project (VIP) value ≥ 1, a total of 1037 differential metabolites (DMs) were identified among the annotated 3322 metabolites. There were 399 up-regulated and 638 down-regulated among these differentially expressed metabolomics (DEMs) between Brazilian (H) vs Df19 (S) (**Supplementary Table S6**). The top-10 up-regulated metabolites in Df-19 were 3-O-Feruloylquinic acid, Cis-5, 8, 11, 14, 17-Eicosapentaenoic Acid, DIBOA-glucoside, Trans-Crocetin (beta-D-glucosyl) (beta-D-gentibiosyl) ester, Rhodojaponin V, Red chlorophyll catabolite, L-Valyl-L-Leucine, Holyrine A, Cephaeline and Prim-O-glucosylcimifugin. The top-10 down-accumulated metabolites in Df19 were Erbstatin Analog, 5-Hydroxy-6-methoxy-3’,4’-methylenedioxyfurano[2”,3”:7,8] flavanone, N-Acetyl-O-demethylpuromycin-5’-phosphate, Myricanone, Valienone, 1-O-Feruloyl-3-O-p-Coumaroylglycerol, Nocardicin G, gamma-L-Glutamyl-L-Glutamic acid, (Z)-3-Peroxyaminocrylate and Kaempferol 3-sophorotrioside (**Figure 7A**). We further functionally annotated the differentially accumulated metabolites (DAMs) in KEGG database and found that the most significantly enriched pathways were ABC transporters, isoquinoline alkaloid biosynthesis, purine metabolism, phenylalanine metabolism, amino sugar and nucleotide sugar metabolism, tryptophan metabolism, phenylpropanoid biosynthesis, Phenylalanine, tyrosine and tryptophan biosynthesis and Glycine, serine and threonine metabolism (**Figure 7B**). The Differential metabolite KEGG enrichment network diagram indicates that metabolites are related in five pathways, namely ABC transporters, amino sugar and nucleotide sugar metabolism, Glycerophospholipid metabolism, Lysine degradation and phenylalanine metabolism (**Figure 7C**).

### Combined transcriptome and metabolome analyses

**Figure 8:**
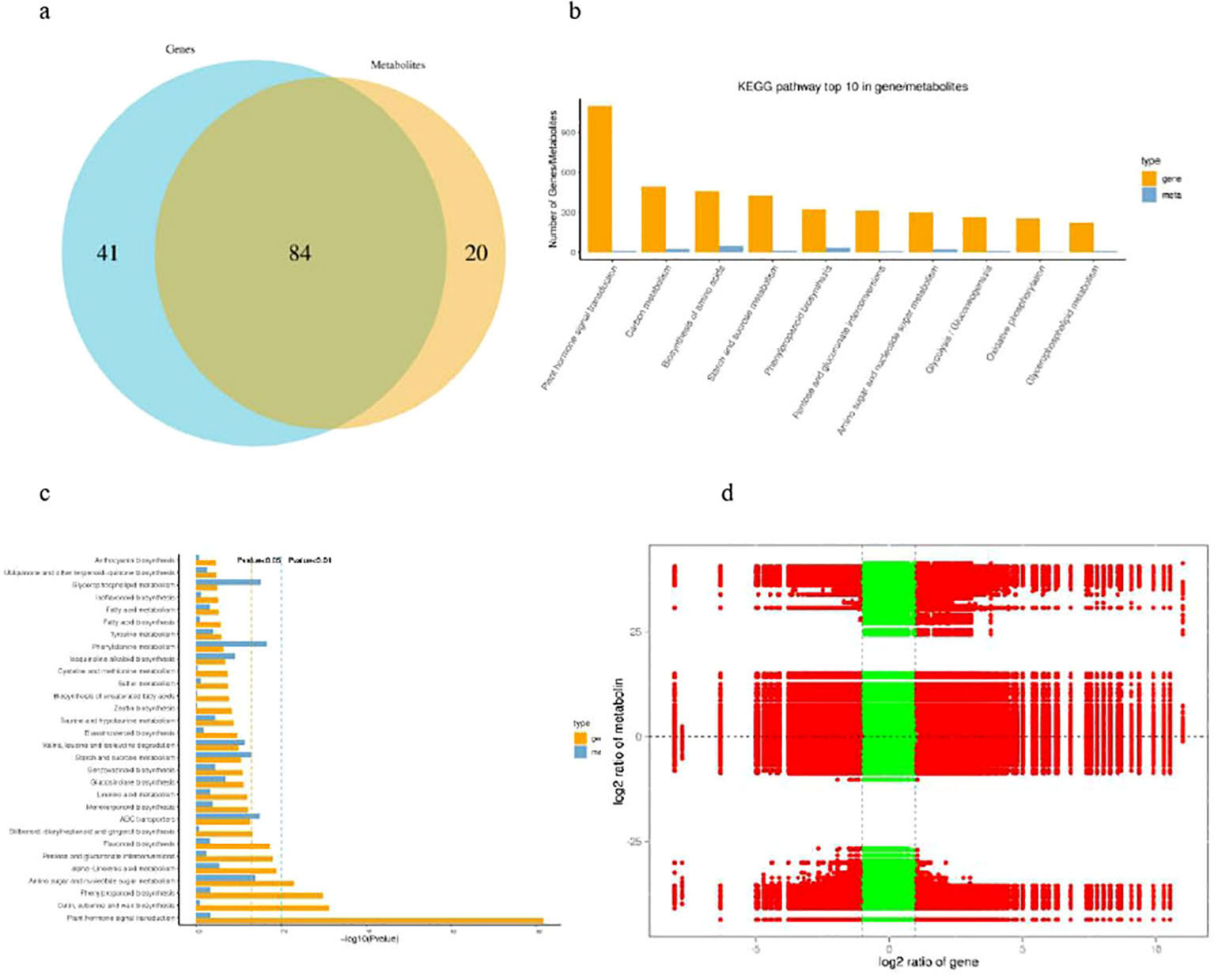
Joint analysis of the differentially expressed genes and differentially accumulated metabolites of banana between H vs S. **(A)** Veen diagram of differential genes and differential metabolite pathways. **(B)** The top 10 pathways with the most genes/metabolites. **(C)** KEGG pathway enrichement analysis of the differentially expressed genes and differentially accumulated metabolites. **(D)** nine-quadrant graph of the differentially expressed genes and differentially accumulated metabolites.

To establish a relationship between different levels of molecules i.e. transcriptome and metabolome, we first compare the pathways involved by genes in the transcriptome with those involved by metabolites in the metabolome, obtain the number of common pathways involved, and draw a Venn diagram (**Figure 8A**). The top 10 pathways with the most differentially expressed genes/metabolites were plant hormone signal transduction, phenylpropanoid biosynthesis, starch and sucrose metabolism, amino sugar and nucleotide sugar metabolism, pentose and glucuronate interconversions, biosynthesis of amino acids, carbon metabolism, glycerophospholipid metabolism, cysteine and methionine metabolism, glycolysis/gluconeogenesis (**Figure 8B**). KEGG enrichment analysis showed that the DEGs and DAMs were mapped onto eighty three pathways (**Supplementary Table S7**) and the top 30 pathways with significant enrichment of DEGs and DAMs are shown in **Figure 8C** with p-value<0.05 which were the similar based individual enrichment analysis. The nine-quadrant graph was generated based on the screening results of |CC|>0.80 and CCP<0.05 between DEGs and DAMs combined with the difference multiplier (**Figure 8D**). In the nine quadrant diagram, we further observed the third and seventh quadrants and found that the numbers of DAM and DEG were relatively high in these two quadrants, indicating that the differential expression patterns of genes and differential accumulation of metabolites are consistent; The gene and metabolite expression trends in these two quadrants are consistent, indicating a positive correlation between DEG and DAM in these two quadrants, suggesting that DAMs may be regulated by DEGs in these quadrants (**Figure 8C**).

## Discussion

Plant height is one of the important agronomic traits of crops. Ideal plant height is beneficial for mechanized planting and reduces investment costs. Dwarf plants can not only ensure crop biomass while effectively improving crop lodging resistance, but also make full use of land space, improve light energy utilization efficiency, and increase yield per unit area (Hou et al., 2020). Therefore, dwarf breeding is one of the important research directions for cultivating excellent and high-yield varieties. Bananas are an important major cash crop widely grown in tropical and subtropical regions, especially in developing countries. However, commercial banana varieties are tall plants exceeding two meters in height, and their high tree crowns make them vulnerable to poor adaptability and severe damage caused by typhoons and storms, causing irreparable damage to commercial plantations. Dwarf bananas have the advantages of efficient agronomic measures such as high-density planting, timely cultivation, and fruit harvesting (Dash and Rai, 2016). Therefore, identifying and utilizing genes related to banana dwarfism is of great significance for cultivating dwarf banana varieties. Dwarf plants are useful materials for exploring and studying genes related to dwarfism. In this study, Df19 is a local dwarf banana variety. Based on our years of observation and measurement results, compared to Brazilian banana plants, Df19 is stronger, has shorter fruits, and exhibits dwarfing characteristics. Therefore, we used the widely planted varieties Brazilian banana and local dwarf banana (Df19) as experimental materials, aiming to identify differentially expressed genes and metabolites related to plant height between Brazilian banana and local dwarf banana (Df19) through transcriptome sequencing analysis, metabolome sequencing analysis, combined with phenotype indicators and hormone measurement analysis. Transcriptome sequencing analysis studies have shown that a total of 2851 DEGs were identified with 1690 upregulated genes and 1161 downregulated genes between Brazilian (H) and Df19 (S). The results of changes in gene expression indicate that height of banana plants may be influenced by factors such as ethylene, hevamine, glycosyl hydrolase, esterase/lipase,endo-1,4-beta-mannosidase. GO enrichment analysis result suggested that these DEGs might occupy an important position in the molecular mechanism of height of banana plant. The differential expressions of jasmonic acid-related genes may be one of the main reasons for the height difference between Brazil (H) and Df19 (S) plants.KEGG Pathway analysis of DEGs indicated that these DEGs genes mainly belong to plant hormone signal transduction, Cutin, suberine and wax biosynthesis, Phenylpropanoid biosynthesis, MAPK signaling pathway-plant, Amino sugar and nucleotide sugar metabolism, Protein processing in endoplasmic reticulum, alpha-Linolenic acid metabolism, Pentose and glucuronate interconversions, Flavonoid biosynthesis, Plant-pathogen interaction, Stilbenoid, diarylheptanoid and gingerol biosynthesis, ABC transporters, Monoterpenoid biosynthesis, Linoleic acid metabolism, Fatty acid elongation, Glucosinolate biosynthesis, Benzoxazinoid biosynthesis, Starch and sucrose metabolism. Among the pathways significantly enriched in DEGs, plant hormone signal transduction was the largest complex.

Plant growth and height are complex processes influenced by factors such as external environment, hormones and genetic factors. The plant height trait is not only controlled by internal genes, but also influenced by various hormones and external environmental factors (Busov et al., 2008; Zhang et al., 2020; Chen et al., 2020). Plant hormones play an important role in plant growth and development. Plant hormones, as the main endogenous signal, can respond quickly to external stimuli received by plants. Research has found that Gibberellin (GA), brassinosteroid (BR) and indole-3-acetic acid (IAA) have significant effects on plant height. In addition, during plant development, these hormones also interact with ethylene, jasmonic acid and Salicylic acid (SA) (Spielmeyer et al., 2002; Deng et al., 2021, 2022). Based on the KEGG enrichment analysis and determination of plant hormone content in leaves, we further analyzed and studied the DEGs related to plant hormone signaling pathways between Brazil (H) and Df19 (S) in bananas, including auxin, cytokinin, gibberellin, abscisic acid, ethylene, oleagin, jasmonic acid, and salicylic acid. The dwarf phenotype resulted from the changes in both cell division and cell elongation, which are regulated by endogenous cytokinin, gibberellin, auxin, abscisic acid and brassinosteroid (Zheng et al., 2019). Among these key plant hormones, auxin is considered one of the most important hormones in the mechanism of dwarfism. Auxin is a small organic acid that has a significant impact on cell division, elongation, and differentiation in higher plants which plays a crucial role in various cellular and developmental responses throughout the entire life cycle of plants (Ma et al., 2016). Plants can quickly perceive and respond to changes in auxin levels, which involve several major types of auxin responsive genes, including the auxin/indole-3-acetic acid (Aux/IAA) family, auxin responsive factor (ARF) family, small auxin upregulated RNA (SAUR), and auxin responsive Gretchen Hagen3 (GH3) family. Aux/IAA proteins are well-known early auxin responsive proteins that participate in auxin signaling by interacting with ARF proteins as transcriptional repressors (Li et al., 2016).The Aux/IAA family members play a crucial roles in plant development including root system development, aboveground growth and fruit ripening etc ( (Ljung, 2013). Cai et al. identified seven genes involved in the IAA signaling pathway from modules positively correlated with plant height using WGCNA which indicates that IAA biosynthesis was involved in regulating plant height development and believed that the auxin response factors were important for the positive regulation of plant height (Cai et al., 2023). Tryptophan is an important precursor substance for plant auxin biosynthesis. The GH3 acylamide synthase family plays a crucial role in the binding of IAA to acyl-containing small molecule substrates (such as amino acids and jasmonic acid) that regulates plant growth and stresses by regulating auxin homeostasis (Luo et al., 2023). SAURs is a class of small auxin up-regulated RNAs. The expression changes of these key genes involved in the auxin signaling pathway may be a component of reduced auxin content in dwarf plants. The roles of cytokinin and IAA in regulating cell elongation are mutually antagonistic. Cytokinin seem to primarily regulate root growth by altering the distribution and sensitivity of auxin, which in turn regulates cell division hormones to maintain appropriate balance (Chaiwanon et al., 2016). In higher plant the cytokinin signaling pathway is mainly mediated by AHKs, AHPs and ARRs. AHKs are histidine kinases including AHK2, AHK3, and AHK4 (also known as CYTOKININ RESPONSE1 (CRE1) or WOODENLEG 1(WOL) as cytokinin receptors that upon binding of cytokinins initiate a phosphorylation signaling cascade that leading to the phosphorylation and activation of specific response regulatory factors (Svolacchia and Sabatini, 2023).The ARRs family is a response regulatory factor activated in response to cytokinins which can be divided into two main subgroups: A-ARRs and B-ARRs. B-ARRs are transcription factors that positively regulate cytokinin responses and are the main response genes for cytokinin inducing. And A-ARR is a negative regulator of cytokinin signaling. A-ARR and B-ARR are both large gene families expressed in different organs and plant tissues. AHPs act in multistep phosphorelay signaling pathways. The phosphoryl transfer from the histidine of AHPs to the aspartate of the N-terminal receiver domain (RD) of B-ARRs enables the DNA binding capacity of the middle DNA binding domain (DBD), thereby allowing a rapid transcriptional response to cytokinin (Zhou et al., 2024).

Gibberellic acid (GA) plays an important role in plant growth and development processes such as seed germination, stem elongation, leaf extension, flower induction, fruit growth and plant elongation (Tyler et al., 2004). Plants with defects in GA synthesis or signal transduction exhibit dwarfism, dark green leaves, delayed growth, and reduced seed yield (Yamaguchi, 2008). GA is one of the most important determinants of plant height and mutations in genes for biosynthesis or signal transduction of GAs generally result in dwarf phenotypes (Sasaki et al., 2002). The deletion of 383 bp in OsGA20ox2 will prematurely terminate the codon, resulting in lower biological activity of GA, and the IR8 sd1mutant plant exhibits a semi dwarf phenotype (Sasaki et al., 2002; Spielmeyer et al., 2002). The GA receptor GID1 can inhibit the inhibitory activity of DELLA on GA signaling (Griffiths et al., 2006). The binding of GA to GID1 drives the interaction between GID1 and DELLA, leading to the degradation of DELLA protein by the 26S proteasome (Davière and Achard, 2013). DELLA belongs to the GARS transcription factor family. When the structure of the protein changes, making it unable to sense GA signals and unable to be degraded, then it can lead to shortened internodes and plant dwarfism (Bolle, 2004). Therefore, GA induced degradation of DELLA protein is considered a key step in the GA signaling pathway. PIF4 is phytochrome-interacting factor 4 and GID1 is gibberellin receptor which inhibit the repression activity of DELLAs on GA signaling (Griffiths et al., 2006). We found that Df19 had the lower levels GA3, while Brazil (H) exhibited the higher GA3 levels. This may be due to changes in some key genes involved in GA biosynthesis and signal transduction in Df19. ABA plays a crucial role in a wide range in regulating plant development processes in plants, especially stress responses to environmental stimuli (Park et al., 2009; Cutler et al., 2010). GA and ABA are widely recognized as important endogenous regulatory factors that play antagonistic roles in plant development and environmental responses. Their interaction is crucial for balancing plant growth and adapting to environmental stress. ABA inhibits GA function by reducing GA biosynthesis and stabilizing the GA repressor DELLA protein (Vanstraelen and Benková, 2012) while GA counteracts ABA signaling by enhancing ABA receptor degradation (Kawa, 2020). Xie et al. found that OsNAC120 plays a crucial role in balancing GA mediated growth and ABA induced rice drought resistance in rice (Xie et al., 2024). The development of plant organs is mainly controlled by the interaction between serine/threonine-protein kinase (SnRK2) and the plant hormone ABA protein phosphatase 2C (PP2C) plays an important regulatory role as a key negative regulatory factor in the activation of ABA signaling (Jung et al., 2020). PP2C inhibits the positive ABA regulator SnRK2 activity, while ABA recognizes PP2C through receptor to releases activated SnRK2 that phosphorylates downstream targets, such as ABFs, and activates ABA response (Cutler et al., 2010). In our study, the differential expression of GID1, DELLAs, PIF4s, PP2C and SNRK2 between Brazilians (H) and Df19 (S) resulted in an increase in ABA content and a decrease in GA3 content, which may be one of the reasons for the dwarfism of Df19.

Ethylene is the smallest plant hormone with a simple C2H4 structure. Ethylene have various functions such as regulating leaf development, senescence, fruit ripening, and stimulating germination, etc. EBF1 and EBF2 are two central F-box proteins that target the major ethylene reactive TFs EIN3 and EIL1 for protein degradation in the absence of ethylene. EIN3 and EIL1 induce the expression of many secondary transcription factors ERF in the presence of ethylene (Potuschak et al., 2003; Nakano et al., 2006). A large number of ethylene-responsive ERFs, including ERF-1, ERF-2 have been proven to be part of a transcriptional network regulating leaf growth inhibition under mild osmotic stress situation (Van den et al., 2017). In young leaves, ethylene and the downstream ERFs emerge as central regulators of leaf growth inhibition, orchestrating both cell division and cell expansion. The accumulation of ethylene in leaves leads to rapid inhibition of cell division and expansion through DELLA mediated mechanisms or through more direct connections with the core cell cycle or EXPANSIN gene (Marieke et al., 2018) Numerous physiological studies have shown that ethylene and gibberellin are involved in the rapid elongation of rice internodes. The ectopic expression of rice AP2/ERF gene (OsEATB) suggests that the crosstalk between ethylene and gibberellin mediated by OsEATB may be the basis for the differences in internode elongation in rice. OsEATB restricts the enhancement of gibberellin reactivity during ethylene induced internode elongation by downregulating the gibberellin biosynthesis gene ent kaurene synthase A (Qi et al., 2011). AtERF11 is a member of the ERF subfamily VIII-B-1a of ERF/AP2 transcription factors in Arabidopsis and is a novel positive regulator of both GA biosynthesis and GA signaling for internode elongation. AtERF11 plays a dual role in promoting internode elongation by inhibiting ethylene biosynthesis and activating GA biosynthesis and signaling pathways (Zhou et al., 2016). In our study, the differential expression of SIMMKK, EIN2, EIN3,EBF1/2 and ERF1/2 between Brazilians (H) and Df19 (S) which may be one of the reasons for the dwarfism of Df19.

Brassinolide (BR) is a natural plant hormone widely present in organs such as pollen, seeds, stems, and leaves of plants and regulate plant growth, differentiation, and homeostasis which sensed by membrane localized receptor kinases (Wang et al., 2001). Defects in BR biosynthesis or signal transduction can lead to serious growth defects, including dwarfism, infertility, and photomorphogenesis in the dark (Li et al., 1996; Clouse et al., 1996); Li and Chory, 1997). BRI1 is a leucine-rich repeat (LRR) receptor kinase, which is a key component of the BR receptor (Wang et al., 2001). BRI1 senses BR signals through its extracellular domain and initiates a signaling cascade through its cytoplasmic kinase activity. BIN2 is a glycogen synthase kinase 3-like protein that functions as a negative regulator of BR response (Li and Nam, 2002). BZR1 is a positive regulator of the BR signaling pathway, playing a role in the feedback regulation of BR induced growth response and BR biosynthesis (Wang et al., 2002). BR and GA act interdependently through a direct interaction between the BR-activated BZR1 and GA-inactivated DELLA transcription regulators. GA promotes cell elongation through BR signaling while BR or active BZR1 inhibits the GA deficient dwarfing phenotype. DELLA directly interacts with BZR1 and inhibits BZR1-DNA binding *in vitro* and *in vivo* (Bai et al., 2012). Previous studies have shown that hormonal signals such as IAA, GA and BR transcription factors interact with each other to promote and regulate stem elongation (Santner and Estelle, 2009). BR can promote the transport of IAA, IAA can promote the expression of the BR synthesis protein DWF4, and both IAA and BR signals can promote GA biosynthesis (Lucas and Prat, 2012). In our study, the plant hormone content test showed that the BR of Df19 (S) was 24.91% higher than Brazilian (H). The expression of BRK1 were up-regulated and the expression of BZR1/2 were down-regulated between Brazilian (H) vs Df19 (S).

JA participates in the regulation of plant growth and development, as well as stress and defense responses.The crosstalk between BR and JA signaling is equally crucial for plant growth and defense responses. The rice Glycogen synthase kinase3 (GSK3)-like kinase OsGSK2 is a conserved kinase serving as a key suppressor of BR signaling which enhanced antiviral defense and the JA response by activating JA signaling as it directly interacts with phosphorylates, and destabilizes OsJAZ4 (He et al., 2020). In our study, it was revealed that the expression of four JAZ were up-regulated and thirteen JAZ were down-regulated, the expression of five MYC2 transcription factor were up-regulated and four MYC2 transcription factor were down-regulated between Brazilian (H) vs Df19 (S). Salicylic acid (SA) plays a role in plant growth and development, photosynthesis, transpiration, as well as ion absorption and transport (An and Mou, 2011). SA and JA are both the main participants in plant immunity. Many studies have confirmed that signal interactions mediated by SA and JA coordinate plant immune responses to pathogens. TGA are essential to the growth and development of plant organs, including roots, stems, leaves, flowers, and fruit (Lu et al., 2024). NPR1 is the main core factor of SA signal transduction, and it interacts with the TGA transcription factor family, possibly serving as a transcriptional co activator of SAR gene expression (Cao et al., 1997). In our study, the expression of TGA were up-regulated between Brazilian (H) vs Df19 (S) which may benefit banana plant growth.

As upstream regulatory factors of metabolic pathways, transcription factors play a crucial role in gene expression (Shan et al., 2013). Here, we identified some transcription factor family members genes consist of MYB, bHLH, AP2/ERF-ERF, NAC and so on between Brazilian (H) and Df19 (S). Many transcription factors are important regulators of plant height. WRKY are one of the largest families of transcription regulatory factors in plants, and are components of signal networks that regulate many plant processes (Rushton et al., 2010).WRKY46/54/70 can be activated by BR and is a cofactor for BES1 to regulate BR target genes (Chen et al., 2017). A rice R2R3-type Myeloblastosis (MYB) transcription factor MYB110 can regulates plant height, lodging resistance, and grain yield in rice (Wang et al., 2024). The BES1/BZR1 family is a group of plant specific transcription factors with non-standard bHLH domains. The members of this family are key transcription factors that regulate the expression of BR response genes. However, the BES1/BZR1 family transcription factors also regulate several aspects of plant development through BR independent pathways (Shi et al., 2022). These transcription factors genes may contribute to study the molecular mechanism of banana plant growth. In this study, we also found that ATP binding cassette (ABC) transporters are one of the significantly enriched pathways in the transcriptome and metabolome. The ABC superfamily is a large, extensive, and diverse protein population that is widely present in organisms, particularly prominent in plants. ABC transporters have been shown to function not only as ATP dependent pumps, but also as ion channels and channel regulators. ABC transporters play an important role in auxin transport, transporting compounds on the cell membrane and participating in various biological processes, most of which mediate transmembrane transport. Plant hormone auxin must be transported in plants in a cell to cell manner to affect its various physiological functions (Theodoulou, 2000). Therefore, our results indicated that the ABC transporters may also play important regulatory roles in collectively regulating the height of banana plants.

## Conclusion

In this study, we used a combination of transcriptome and metabolomics to reveal differentially expressed genes and their related metabolic pathways in tall and short bananas, and analyzed and discussed the molecular mechanisms controlling banana height by combining morphological differences and endogenous hormone content. Our analysis results confirm that the dwarfism phenotype is the result of the synergistic effects of hormones such as ABA, GA3, IAA, JA, BR and other plant hormones related to growth. In addition, transcription factors and ABC transporters may also play important regulatory roles, collectively regulating the height of banana plants.

## Data Availability

The datasets presented in this study can be found in online repositories. The names of the repository/repositories and accession number(s) can be found in the article/**Supplementary Material**.
