# Supplementary figures and images for: Comprehensive analysis of morphology, transcriptomics, and metabolomics of banana (Musa spp.) molecular mechanisms related to plant height
Source: Front Plant Sci. 2025 Mar 25;16:1509193. doi: 10.3389/fpls.2025.1509193 (PMC11975952; doi:10.3389/fpls.2025.1509193)

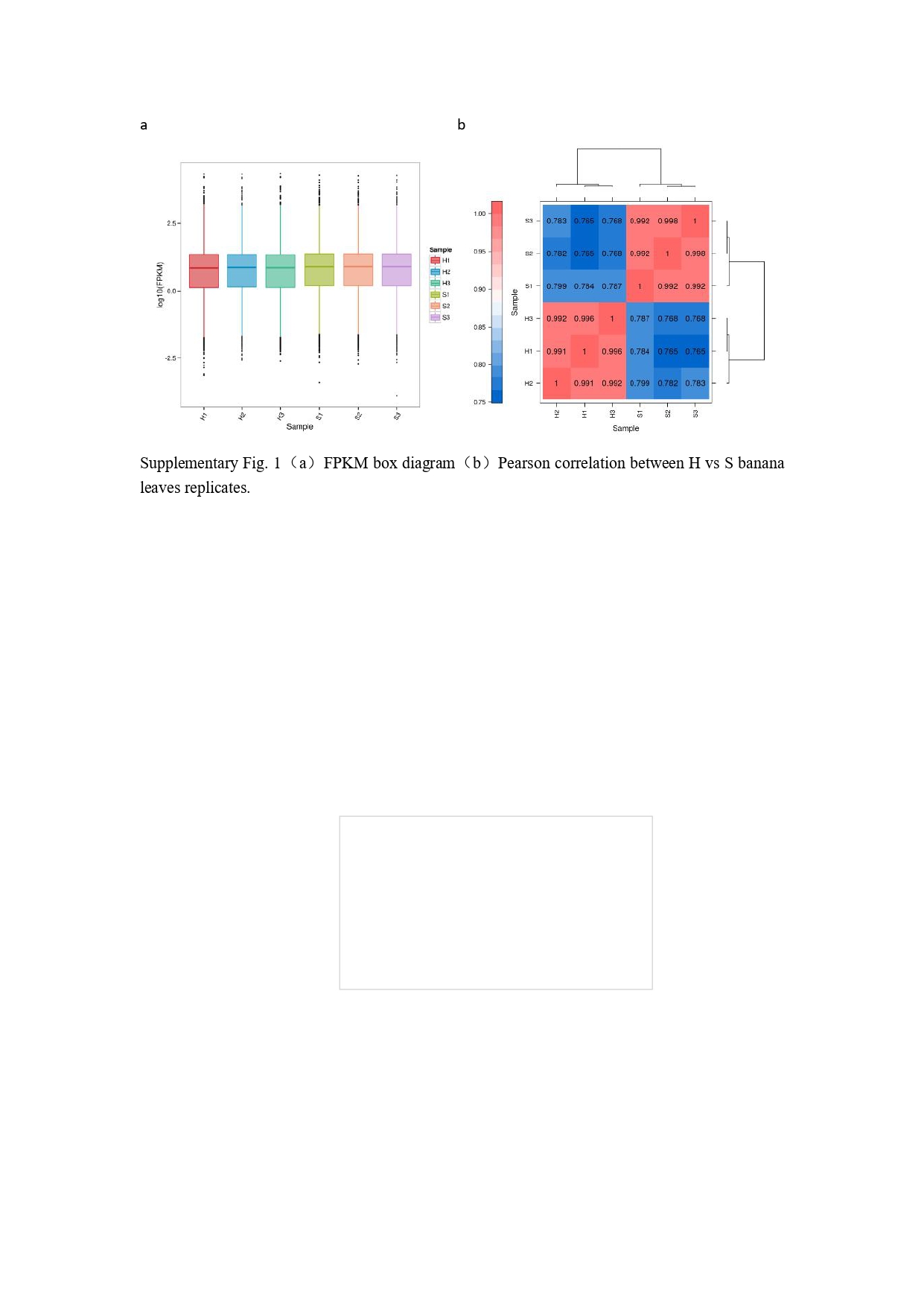

Supplement: Supplementary file 1 [file Image1.jpeg]

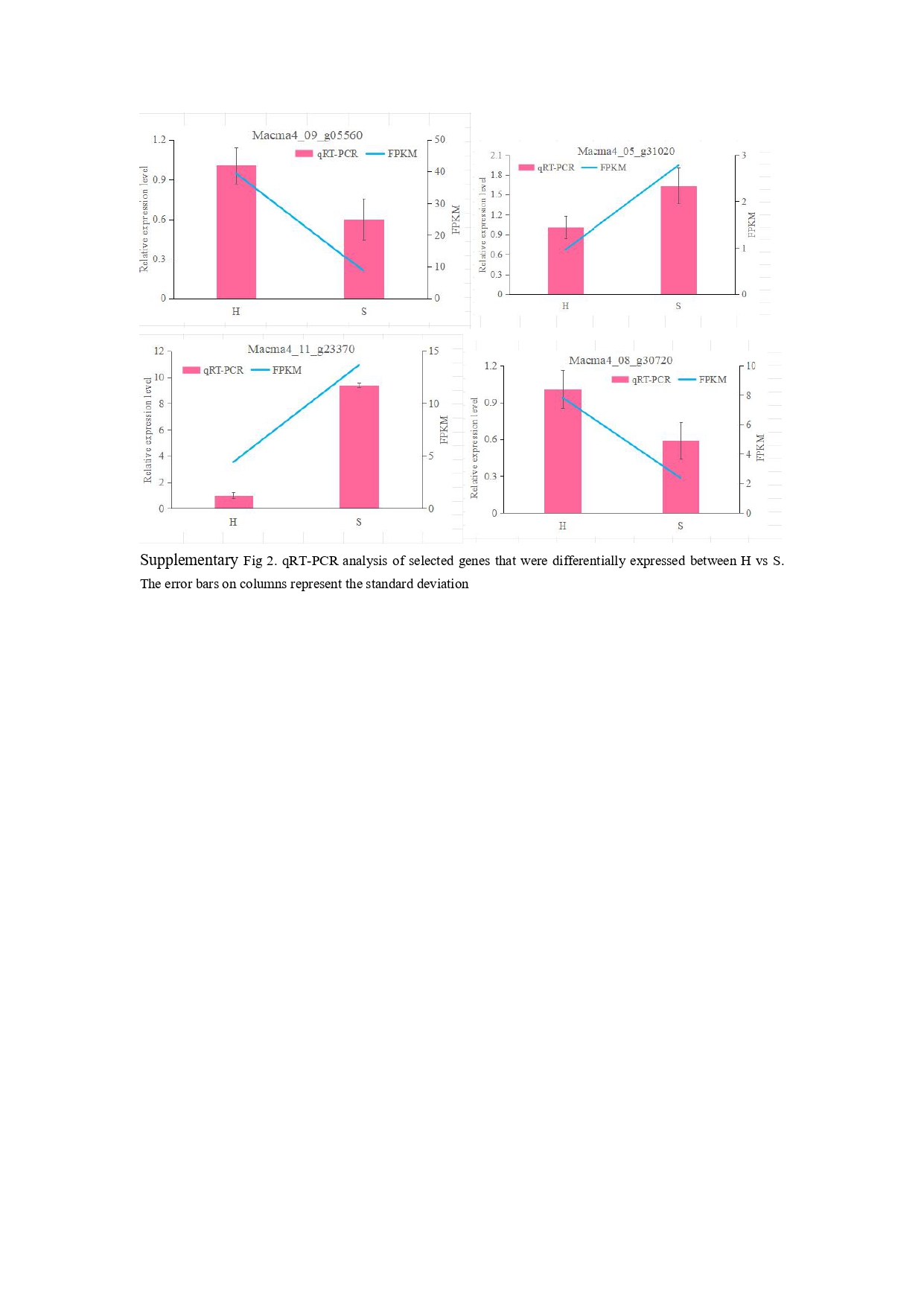

Supplement: Supplementary file 2 [file Image2.jpeg]

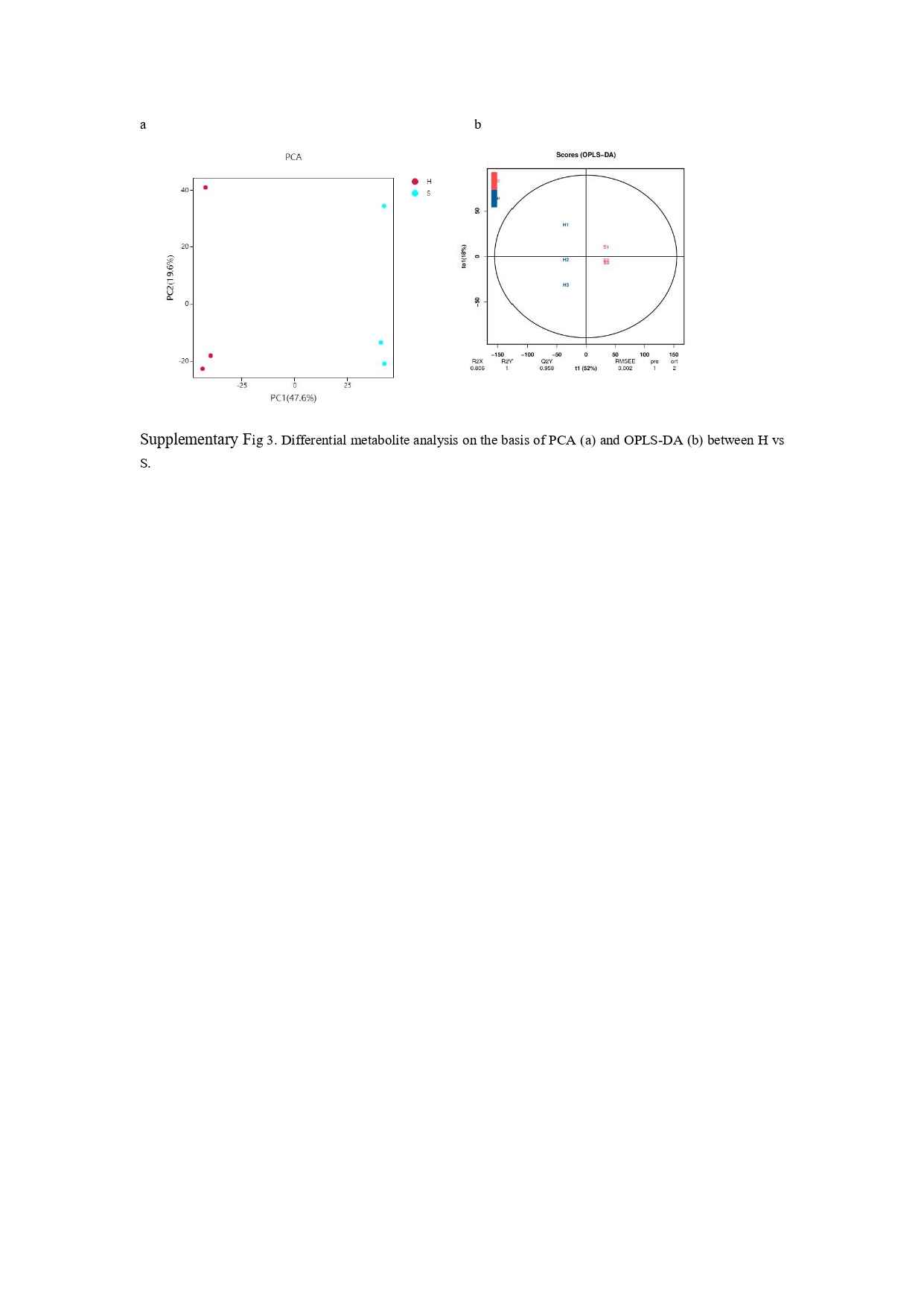

Supplement: Supplementary file 3 [file Image3.jpeg]
